# Measuring perceived legitimacy of food quality labels: A Mixed qualitative-quantitative approach for developing formative indicators^☆^

**DOI:** 10.1016/j.mex.2026.103920

**Published:** 2026-04-20

**Authors:** Maria Bouhaddane, Rafia Halawany-Darson, Corinne Rochette, Corinne Amblard

**Affiliations:** a Université Clermont Auvergne, INRAE, VetAgro Sup, UMR 545 Fromage, 63370 Lempdes, France; b Université Clermont Auvergne, CleRMa, IAE Clermont Auvergne, 11, Boulevard Charles de Gaulle 63000 Clermont-Ferrand, France

**Keywords:** Legitimacy, Scale proposition, Questionnaire, Food labelling, Consumer, Mixed approach

## Abstract

Legitimacy is defined as the generalized perception that an entity’s actions are appropriate within a socially constructed system of norms, values, and beliefs. While this concept is well established in organizational literature, consumer perceptions of food quality labels’ legitimacy remain underexplored. To address this gap, we adapted the organizational legitimacy framework—cognitive, regulative, pragmatic, and moral dimensions—to the context of quality labels and developed a corresponding measurement instrument.

Perceived legitimacy was conceptualized as a second-order formative construct composed of four first-order formative dimensions. Using a mixed qualitative-quantitative approach, we designed and validated indicators for each dimension. Our methodology involved:

• Qualitative exploration to generate indicators aligned with each legitimacy dimension.

• Quantitative validation through a survey of 600 French consumers.

• Partial Least Squares (PLS) modeling to test reliability and validity.

This article details the development process and validation of the proposed instrument. Despite some contextual limitations, the model offers a novel framework for understanding the multifaceted nature of legitimacy in consumer evaluations of labels. The results confirm the relevance of legitimacy as a construct in label studies and provide useful insights for consumer behavior research. The method can be replicated in other labeling or geographical contexts.

## Graphical abstract

**Figure:**
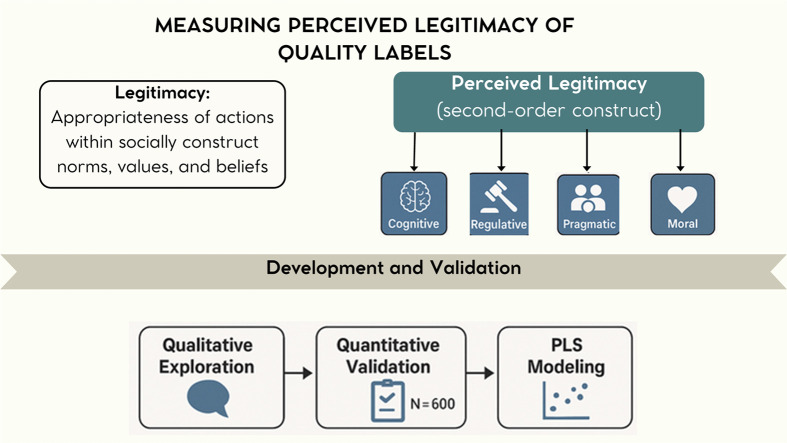

**Specifications table**

**Table:** 

**Subject area**	*Economics and Finance*
**More specific subject area**	*Social Sciences*
**Name of your method**	*Measuring perceived legitimacy of food quality labels: A Mixed qualitative-quantitative approach for developing formative indicators*
**Name and reference of original method**	*Diamantopoulos, A., & Winklhofer, H. M. (2001). Index construction with formative indicators: An alternative to scale development. Journal of Marketing Research, 38(2), 269–277.*
**Resource availability**	• *Software supporting qualitative data analysis (*e.g.*, NVivo)* • *Statistical software for structural equation modelling (*e.g.*, SmartPLS)*

## Background

Quality labels and standards have been shown to enhance consumer preferences and willingness to pay for food products, as they serve as quality signals [[1], [2], [3], [4], [5]]. However, the proliferation of such labels contributes to consumer confusion, potentially undermining their intended purpose. Despite their widespread use in agri-food markets, the legitimacy of these labels remains contested [6], particularly due to well-publicized controversies over potential misuse and the opacity of self-proclaimed commercial claims. In this context of information overload and label inflation, legitimacy emerges as a crucial analytical lever for understanding how consumers interpret and respond to certification schemes.

Legitimacy is often defined in the organizational literature as *“the generalized perception or assumption that the actions of an entity are desirable, proper, or appropriate within some socially constructed system of norms, values, beliefs, and definitions”* [7]. As a key driver of consumer trust [[8], [9], [10]], perceived legitimacy shapes both product evaluations [11] and purchase intentions [12]. Yet, while the concept of legitimacy is well-established in organizational theory, its application in consumer research—particularly in the context of food labeling—remains underexplored.

Most existing research has framed legitimacy either as a property or a process, with numerous studies using proxy variables to measure the degree of legitimacy held by an organization or industry [13]*.* Recent efforts have sought to operationalize legitimacy by developing instruments that capture individual-level perceptions (e.g., [14]). However, these studies have primarily focused on organizations like banks or retail chains [15], offering valuable insights but failing to account for the *idiosyncratic features* of quality labels. Moreover, empirical research in this area has predominantly focused on the operationalization of Suchman’s [7] typology—pragmatic, moral, and cognitive legitimacy—while the regulative dimension has received comparatively little attention. Yet, for quality labels, regulatory legitimacy is particularly important, as it encompasses stakeholders’ evaluations of governance structures, the rigor of rule-setting procedures, and the impartiality of compliance mechanisms.

This gap in the literature calls for a deeper investigation into how consumers define and assess the legitimacy of quality labels. To address this Bouhaddane et al. [16], proposed a conceptual model to examine the impact of perceived label legitimacy —using the Protected Designation of Origin (PDO) label as a case— on perceived product quality and purchase intention, and developed a measurement instrument to capture the perceived legitimacy construct. Building on this work, the present study operationalizes perceived legitimacy from the consumer’s perspective for food quality labels by integrating four dimensions (cognitive, regulative, pragmatic and moral) into a second-order formative construct. This paper details the mixed-methods approach used to develop and validate this instrument.

Whereas the majority of legitimacy measures rely on reflective, perception-based psychometric scales grounded in Suchman’s [7] tripartite typology and are primarily designed to capture stakeholders’ evaluations of focal organizations (e.g., [14,17]), our approach departs from this tradition in two key respects. First, legitimacy is examined at the level of a market device (i.e., a quality label) rather than an organization. Second, perceived legitimacy is conceptualized as a formative construct, defined by its indicators rather than reflected by them. This specification makes it possible to identify the concrete elements that jointly construct legitimacy perceptions. The method further extends existing approaches by systematically integrating regulative legitimacy and by combining consumer evaluations with institutional attributes (e.g., governance procedures, control mechanisms, and formal specifications) within a single framework. By capturing both the attitudinal and structural foundations of legitimacy, the proposed instrument is particularly suited to quality labels, whose legitimacy is rooted not only in beliefs but also in formal regulatory arrangements. This positioning aligns our method with recent integrative measurement frameworks [18], while providing a multidimensional and replicable framework that can be adapted to other labelling schemes or geographical contexts.

More broadly, this contribution responds to calls for context-specific measurement tools that account the socio-cultural and institutional embeddedness of consumer perceptions. It also highlights the relevance of formative measurement models for capturing complex, multidimensional constructs such as legitimacy, which are socially constructed and defined by their dimensions and indicators rather than reflected by them.

## Method details: a mixed qualitative-quantitative approach for developing formative measurements

### The choice of a formative model for the perceived legitimacy construct

Constructs are not inherently reflective or formative [19]; rather, the decision to model a construct as reflective or formative should be guided by theoretical foundations, the study’s objectives, and empirical considerations [20,21]*.* In the case of legitimacy, the literature mentions the use of both types of measurement approaches.

In this study, we adopt a formative conceptualization of perceived legitimacy, aligned with our primary research objective: to understand how consumers construct perceptions of label legitimacy and to identify its underlying components. Formative measurements are represented as *" defining characteristics that collectively explain the meaning of the construct "* ([22], p. 713)*.* In this sense, the indicators are not outcomes of the construct, but rather its building blocks.

While a reflective measure would assess whether a label is perceived as legitimate, a formative model allows us to identify the specific factors contributing to this perception. This is particularly relevant for a multidimensional concept such as legitimacy, which encompasses cognitive, regulatory, pragmatic, and moral dimensions. A formative approach enables us to capture the contribution of each of these facets to the overall perception of legitimacy. Accordingly, we conceptualize perceived legitimacy as a second-order formative-formative construct: the global perception of legitimacy is formed by its four dimensions, each of which is itself built from a distinct set of formative indicators.

Unlike reflective scale development—where detailed methodological guidelines exist for construct specification, item selection and purification, and scale validation (e.g., [23,24]) - the development of formative measures remains less standardized. Based on a set of critical issues identified in the literature on formative indicators, Diamantopoulos and Winklhofer [21] proposed a four-stage protocol to ensure a successful index construction:

1. **Content specification:** defining the conceptual domain of the construct by identifying its general components;
2. **Indicator specification:** conducting a literature review and semi-structured interviews to identify indicators needed that cover all relevant facets of the construct;
3. **Indicator collinearity analysis:** identifying and removing indicators with high collinearities to assess the specific influence of each indicator;
4. **External validity analysis:** evaluating the relevance of each indicator and removing those that do not contribute meaningfully.

Based on this framework, we implemented a four-step abductive process to develop measures for the four dimensions of perceived legitimacy. This process involved: (1) defining the construct domain; (2) generating indicators; (3) refining measures through expert consultation; and (4) conducting validity analyses. To assess external validity, a structural model was developed, incorporating both antecedents and outcomes of the perceived legitimacy construct.

**Step 1: Definition of the conceptual domain of the perceived legitimacy construct**

Defining the conceptual domain of a construct is a critical step in delineating the content it is intended to represent and in guiding the measurement approach. This process is key to ensure the construct’s content validity [25], particularly in the case of a formative construct, which is formed by its indicators—rather than reflected by them—making the specification of indicators inseparable from the definition of the construct itself [21].

Given that formative indicators collectively define the construct’s meaning, the omission of relevant indicators may alter its conceptual nature and empirical interpretation [19,22,26]. The construct domain must therefore be specified as exhaustively as possible to ensure an accurate representation of the focal concept [21].

**Fig. 1:**
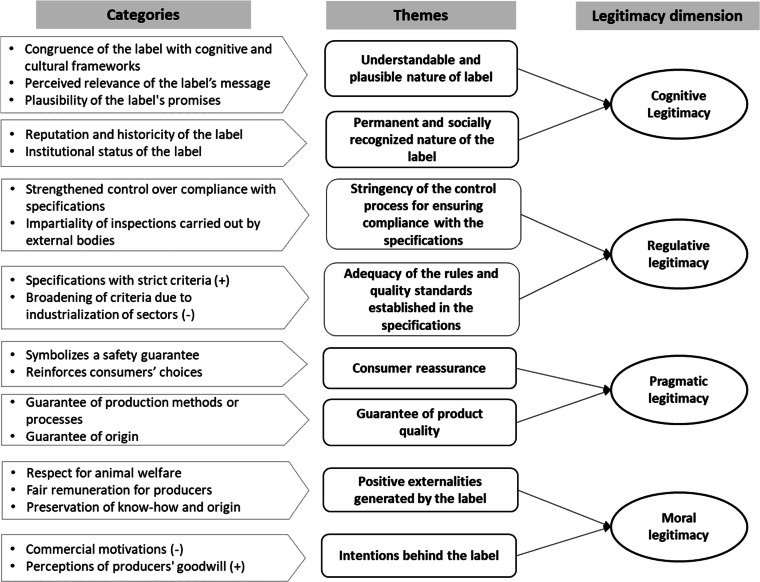
Thematic coding tree from the qualitative study.

*Source:* Authors.

In specifying the domain of the perceived legitimacy construct, we drew upon the theoretical frameworks proposed by Suchman [7], Scott [27], and Deephouse et al. [28], which together informed the identification of four key dimensions: cognitive, regulative, pragmatic, and moral. Furthermore, within the scope of our research, insights from 11 semi-directed interviews with consumers contributed to the delineation of the conceptual domain of perceived legitimacy in the specific context of quality labels. A content analysis of respondents’ discourse revealed key consumer expectations regarding labeling schemes and clarified the evaluative criteria underpinning each of the four dimensions of legitimacy, as illustrated in Fig. 1. The definitions adopted for each dimension are presented in Table 1.

**Table 1:** Definition of the conceptual domain of perceived legitimacy dimensions.

Dimensions of the perceived legitimacy	Definition
*Cognitive legitimacy*	It reflects the obvious, self-evident or "taken-for-granted" nature of a label [[7], [14]] -that is, the familiarity it acquires as it becomes institutionalized [29]. It therefore depends on the label’s history [30] and its potential to be socially accepted as both necessary and enduring [[7], [31]].
*Regulative legitimacy*	It emerges from compliance with the law or other forms of collective regulation (Greenwood et al., 2002; Greve, 2005; [27]). According to Scott ([27], p. 42), it is defined as the degree of adherence to "explicit regulatory processes: rule-making, monitoring and sanctioning activities". In the specific case of labels, the evaluation of regulatory legitimacy focuses on the procedures for establishing and regulating the criteria outlined in the specifications and, more generally, on the governance structures and operational rules specific to each label.
*Pragmatic legitimacy*	It considers the consumer's own interests [7] and is based on an assessment of the value and benefits the label provides to consumers [14]. Assessing the pragmatic legitimacy of a label means examining its ability to fulfill its purpose, that is satisfying consumers' expectations and needs in terms of information, reassurance and quality assurance.
*Moral legitimacy*	It results from an evaluation based on the benefits it brings to society, and thus reflects the societal value and worth attributed to it [27]. It stems from the extent to which the objectives and outcomes that the label helps to achieve are in line with socially constructed norms and values, thereby contributing to promote societal well-being [[7], [14]].

**Step 2: Development and selection of the perceived legitimacy indicators**

Building on the conceptual definitions established in Step 1, we generated an initial pool of indicators for each dimension of perceived legitimacy. These formative indicators were primarily derived from the results of our qualitative study (summarized in Fig. 1), allowing us to capture the specific characteristics of quality labels and go beyond the conventional boundaries of legitimacy measures found in the organizational literature. Where possible, we incorporated participants’ own wording to align more closely with consumer language and perceptions. This initial set was then supplemented by a review of existing legitimacy measures. As a result, seven items were adapted or directly adopted from validated scales, as detailed in Table 2. In total, 17 indicators were compiled and categorized according to the four components of perceived legitimacy, based on our literature review and qualitative findings.

**Table 2:** Initial pool of indicators measuring the perceived legitimacy of a quality label.

Construct	Indicator	Adapted from
**Cognitive Legitimacy**	CL1. I think that this label today is a continuation of what it was before.	Capelli & Sabadie [32]; Lapeyre [10]; Lichtlé et al. [9]
	CL2. This label conveys clear, comprehensible information.	
	***CL3. I have a positive opinion about this label.***	Chung et al. [17]
	***CL4. The general public approves of the use of this label.***	Elsbach (1994)
**Regulative Legitimacy**	RL1. I associate this label with rigorous quality control.	
	RL2. This label is controlled by independent bodies.	
	***RL3. This label follows government regulations.***	Elsbach (1994); Chung et al. [17]
**Pragmatic Legitimacy**	PL1. This label makes it easier for me to find information and provides me with a reference point.	
	***PL2. This label is informative (*Vs *without input).***	Capelli & Sabadie [32]
	PL3. This label confirms my choice.	
	***PL4. This label informs me of the superior quality of the product.***	
	PL5. I see this label as a guarantee of compliance with production standards and criteria.	
**Moral Legitimacy**	ML1. This label is designed to better inform consumers (Vs. is used as a means of advertising persuasion).	Lichtlé et al. [9]
	ML2. This label promotes sustainable consumption.	
	ML3. This label allows for a fairer remuneration of producers.	
	ML4. This label is concerned with meeting acceptable standards of environmental protection, food safety and animal welfare.	Elsbach (1994), Alexiou & Wiggins [14]
	***ML5. This label contributes positively to the society.***	Bitektine et al. [33], Alexiou & Wiggins [14]

To support the validation of the formative measurement model, we also included reflective indicators of perceived legitimacy. In line with the recommendations of Diamantopoulos and Winklhofer [21], these reflective items serve as benchmarks to assess the construct captured by the formative indicators. When possible, they were adapted from established measurement scales used in previous studies [[14], [17], [34]] and are shown in bold italics in Table 2.

**Step 3: Expert consultation and refinement of the perceived legitimacy instrument**

Following the recommendations of DeVellis [24], the initial pool of generated items was subjected to expert evaluation. Four academic experts were invited to contribute to the conceptual refinement of perceived legitimacy as applied to quality labels and to assess the proposed indicators in terms of content and face validity. Each expert received a briefing document outlining the theoretical foundations that guided item development, including definitions of the four dimensions of perceived legitimacy. Experts were asked to evaluate the relevance and conceptual alignment of each item with its intended dimension, and to comment on the clarity and precision of the item wording. These evaluations were followed by individual telephone interviews to allow for in-depth discussion of their assessments.

During these discussions, experts provided valuable feedback on both the conceptual framework and the operationalization of the construct. Their insights led to the addition of several items considered essential to adequately capture the multidimensional nature of perceived legitimacy. In particular, the initial set of items for regulative legitimacy was considered overly focused on compliance and control. To address this limitation, new indicators (RL4 and RL5) were introduced to reflect the intentions and motivations of stakeholders involved in defining the specifications, along with an item emphasizing the importance of compliance within a quality label framework (RL6).

For cognitive legitimacy, three new indicators (CL5, CL6, and CL7) were developed to capture consumers’ perceptions regarding the official status, public reputation, and taken-for-granted nature of quality labels. Additionally, two reflective items (ML8 and RL7) were added for use during the instrument validation phase.

This iterative process of expert consultation and instrument refinement resulted in a final set of 28 indicators (see Table 3; reflective measures are shown in bold italics), enhancing both the robustness and the comprehensiveness of the proposed instrument for measuring perceived legitimacy.

**Table 3:** Final set of indicators measuring the perceived legitimacy of a quality label (following expert consultation).

Construct	Indicator	Changes made following expert assessment
**Cognitive Legitimacy**	CL1. This label has been consistent with its purpose since its inception.	Reformulated to better capture the perceived continuity of the label's purpose over time.
	CL2. This label is comprehensible.	Simplified formulation.
	***CL3. I have a positive opinion about this label.***	
	***CL4. The general public approves of the use of this label.***	
	CL5. This label is an official label.	Added to capture public recognition of the label’s official status.
	CL6. This label has a good reputation among the general public.	Added to reflect the perceived public reputation of the label.
	CL7. Using this label to signal the quality of products to consumers is a given.	Added to capture the taken-for-granted nature of the label as a quality signal.
**Regulative Legitimacy**	RL1. This label is a symbol of rigorous quality control.	Reformulated for improved clarity.
	RL2. This label is controlled by accredited and independent bodies.	“Accredited” added to emphasize legitimacy through oversight by accreditation bodies.
	***RL3. This label follows government regulations.***	
	RL4. This label is based on a set of specifications drawn up by interested and competent parties.	Added to emphasize stakeholder involvement in rule-setting.
	RL5. This label is borne by stakeholders concerned with preserving the authenticity of their production.	Added to highlight stakeholder commitment to authenticity.
	RL6. This label complies with the conditions and requirements specified in the book of specifications.	Added to specify formal compliance with documented standards.
	***RL7. This label complies with production conditions and requirements that go beyond those imposed by public regulations.***	Added as a reflective measure to highlight the label’s elevated voluntary standards.
**Pragmatic Legitimacy**	PL1. This label helps me identify products with the characteristics I am looking for.	Split into two indicators to distinguish informational shortcut from reference status.
	PL4. This label is a reference point for me.	New indicator derived from PL1.
	***PL5. This label conveys clear information about the quality of the product.***	Reformulated to specify the nature of the information provided (i.e., quality).
	PL2. This label reinforces my choice.	Reformulated (“confirms” replaced with “reinforces”).
	***PL6. This label signals a distinctive quality of the product.***	Reformulated (“superior” replaced with “distinctive”).
	PL3. This label provides me with a guarantee of compliance with production standards and requirements.	Reformulated for clarity.
**Moral Legitimacy**	ML1. This label provides transparent communication to consumers about its purpose and objectives.	Reformulated to emphasize perceived transparency regarding the label’s purpose.
	ML2. This label allows for a more sustainable consumption.	Reformulated for improved clarity.
	ML3. This label allows for a fairer remuneration of producers.	
	ML4. This label is concerned with the environment.	Adapted from Elsbach (1994); split into two indicators (ML4 and ML5) to separately address environmental and animal welfare concerns.
	ML5. This label is concerned with animal welfare.	
	ML6. This label contributes to the preservation of landscapes and biodiversity.	Added to explicitly measure perceived contribution to biodiversity and landscape protection.
	***ML7. Overall, this label plays an important role in our society, protecting the interests of both producers and consumers.***	Reformulated and added ML7 to assess the perceived role of quality labels in safeguarding the interests of both producers and consumers.
	***ML8. This label is of benefit to society.***	

**Step 4: Quantitative validation of the perceived legitimacy measurements**

A quantitative validation of the perceived legitimacy measurement instrument was conducted using Partial Least Squares (PLS) modeling, based on a survey of 600 consumers representative of the French population. The study focused on the Protected Designation of Origin (PDO) label, using cheese as a representative food product category. The validation process was carried out at two levels, following the recommendations of Henseler et al. [35]:

- **At the indicator level***,* the validation examined the significance of measurement weights using a bootstrapping procedure [19] and assessed multicollinearity among indicators to ensure the absence of redundancy. Importantly, non-significant weights were not automatically considered problematic. Hair et al. [19] advise complementing weight analysis with an evaluation of indicator loadings, which reflect each indicator’s absolute contribution to the construct, independent of the influence of other indicators. In line with their recommendation, indicators with non-significant weights were retained when their loadings exceeded 0.50.
- **At the construct level**, convergent and nomological validity were tested. Convergent validity was assessed via redundancy analysis, which involves estimating a bivariate model in which the formative construct is used as a predictor of a reflective measure of the same concept [[19], [21]]. Nomological validity was evaluated by examining the significance of the relationships between the formative index and other constructs within a theoretically grounded nomological framework, as supported by prior research [36].

In accordance with the recommendations of various authors (e.g., [[19], [35], [37]]), redundancy analyses were conducted to assess the convergent validity of the formative constructs. In these analyses, each formative measure corresponding to a dimension of perceived legitimacy was modeled as a predictor of a reflective (i.e., global) measure of the same construct. This approach assumes that the reflective measure represents a synthesized judgment, while the formative indicators represent the distinct dimensions contributing to that judgment. Following the recommendations of Chin [38], a coefficient of determination (R²) of at least 0.64, corresponding to a correlation of 0.80 or higher between the formative and reflective constructs, was used as the threshold for establishing adequate convergent validity.

## Method validation

Given the lack of established legitimacy measurements applicable to quality labels and standards, this study aimed to develop a dedicated instrument to measure perceived legitimacy by adapting the organizational legitimacy framework.

To ensure content validity prior to quantitative data collection, we followed the recommendations of Hair et al. [19] and Rossiter [39], confirming the relevance and adequacy of the formative indicators used to represent the construct. The definition and scope of perceived legitimacy were informed by an extensive literature review and insights from a preceding qualitative study. Expert evaluations were then solicited to assess the clarity, relevance, and comprehensiveness of the proposed measurement items.

Quantitative validation was subsequently carried out using Partial Least Squares Structural Equation Modeling (PLS-SEM), supporting the psychometric robustness of the perceived legitimacy construct. The results demonstrated satisfactory reliability, as well as convergent and nomological validity of the formative constructs. As shown in Table 4, the significance of the indicators corresponding to the four legitimacy dimensions—cognitive, regulative, pragmatic, and moral—was statistically confirmed. Variance inflation factors (VIF) for all indicators remained below the threshold of 5, indicating no multicollinearity issues.

**Table 4:** Validity of the perceived legitimacy formative instrument.

	Indicators	Weights	t value	Correlations	VIF
Cognitive legitimacy	*CL3* *CL4* *CL5* *CL6* *CL7*	0443 0185 0154 0174 0278	6939*** 3020*** 2530** 2629*** 4224***	0889 0727 0,74 0792 0791	1990 1646 1812 2109 1763
Regulative legitimacy	*RL2* *RL3* *RL4* *RL5* *RL6*	0318 0191 0346 0197 0143	7297*** 5162*** 7029*** 4408*** 3128***	0851 0774 0885 0809 0807	2045 1887 2425 2143 2322
Pragmatic legitimacy	*PL1* *PL3* *PL5* *PL6*	0190 0406 0396 0162	3031*** 6778*** 8729*** 3046***	0840 0901 0866 0813	2691 2463 1904 2522
Moral legitimacy	*ML1* *ML2* *ML3* *ML4* *ML5* *ML6*	0496 0288 0042 0207 0026 0160	10,101*** 5433*** 0927 *n.s.* 3831*** 0558 *n.s.* 3302***	0888 0835 0656 0763 0656 0728	1838 2040 1720 2431 2027 1970

** (***) Coefficient significant at the 5% (1%) threshold; *n.s.* non-significant coefficient.

VIF = *Variance Inflation Factor.*

**Fig. 2:**
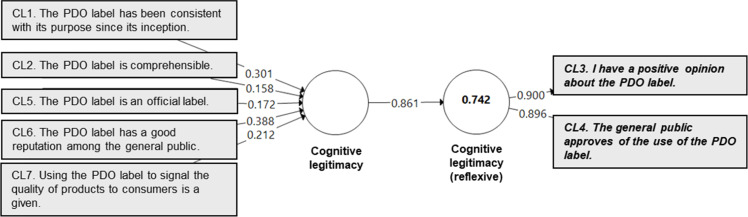
Redundancy model for the cognitive legitimacy construct.

**Fig. 3:**
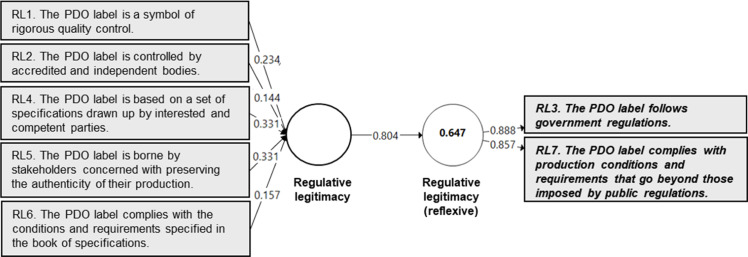
Redundancy model for the regulative legitimacy construct.

**Fig. 4:**
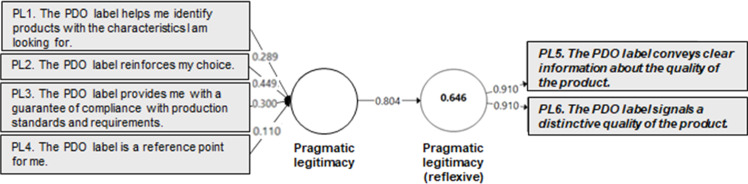
Redundancy model for the pragmatic legitimacy construct.

**Fig. 5:**
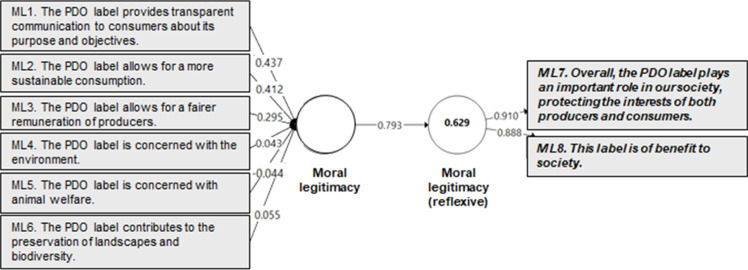
Redundancy model for the moral legitimacy construct.

In addition, redundancy analyses (Fig. 2, Fig. 3, Fig. 4, Fig. 5) confirmed that the formative indicators adequately captured the variance in their respective first-order constructs (R² > 0.64). Taken together, the significance of the indicator weights, acceptable multicollinearity levels, and redundancy results attest to the structural robustness of the proposed measurement instrument. Each legitimacy dimension was effectively represented by its associated indicators, with high loadings and strong predictive relevance. Finally, nomological validity was confirmed within the broader theoretical framework [16], reinforcing the instrument’s credibility and deepening our understanding of how legitimacy influences consumer evaluations and behavior in the context of food labeling.

The proposed method offers a structured and replicable approach for conceptualizing and measuring the perceived legitimacy of food quality labels from the consumer’s perspective. By operationalizing legitimacy as a second-order formative-formative construct composed of four distinct dimensions—cognitive, regulative, pragmatic, and moral—this study extends the application of the legitimacy framework from the organizational domain to consumer behavior and labeling practices.

## Limitations

The following limitations should be interpreted in light of the methodological choices that underpin the originality of the proposed instrument, particularly its formative specification and its strong contextual grounding. The instrument was developed and tested in a specific national and sectoral context—France and the case of food products, particularly PDO-labeled cheeses. While the conceptual framework is theoretically transferable, the indicators were partly derived from qualitative interviews that reflect consumer expectations and vocabulary specific to this context. As such, caution is warranted when generalizing the instrument to other types of quality labels (e.g., organic, fair trade), other product categories (e.g., wines, meats, processed foods), or different cultural and regulatory environments.

Furthermore, although the instrument demonstrated strong psychometric properties, the inclusion of formative indicators limits the applicability of traditional validation techniques such as internal consistency. Researchers aiming to replicate or adapt this method should consider re-evaluating indicator relevance through qualitative inquiry and expert consultation tailored to the new context.

Finally, the cross-sectional nature of the survey design precludes conclusions about the temporal stability of perceived legitimacy. Longitudinal or repeated-measures designs would be necessary to assess how consumer perceptions evolve over time in response to media, regulatory changes, or food-related scandals.

## Ethics statements

*Not applicable.*

## Declaration of competing interest

The authors declare that they have no known competing financial interests or personal relationships that could have appeared to influence the work reported in this paper.

## Data Availability

Data will be made available on request.

## Appendix Supplementary materials

Image, application 1
