# Supplementary material for: Measuring perceived legitimacy of food quality labels: A Mixed qualitative-quantitative approach for developing formative indicators
Source: MethodsX. 2026 Apr 20;16:103920. doi: 10.1016/j.mex.2026.103920 (PMC13136638; doi:10.1016/j.mex.2026.103920)
Supplement: Supplementary file 1 [file mmc1.docx]

**Description of the quantitative sample**

**Table 1.** Sample distribution by respondents’ gender

| Gender | Distribution |
| --- | --- |
| Male | 47,7% |
| Female | 52,3% |

**Table 2.** Sample distribution by respondents’ age

| Age group | Distribution |
| --- | --- |
| 18-24 years | 7,7% |
| 25-34 years | 18,5% |
| 35-44 years | 17,2% |
| 45-54 years | 17,5% |
| 55-64 years | 21,7% |
| 65 years and over | 17,5% |

**Table 3.** Sample distribution by respondents’ household size

| Household size | Distribution |
| --- | --- |
| 1 person | 20,5% |
| 2 persons | 37,2% |
| 3 persons | 17,0% |
| 4 persons | 16,8% |
| 5 persons or more | 8,5% |

**Table 4.** Sample distribution by respondents’ socio-professional category

| Socio-professional category | Distribution |
| --- | --- |
| Farmer | 0,2% |
| Artisan, shopkeeper, or business owner | 4,2% |
| Manager / senior intellectual profession | 13,3% |
| Intermediate profession | 12,7% |
| Employee (public or private sector) | 25,0% |
| Manual worker | 5,8% |
| Retired | 24,2% |
| Student | 4,3% |
| Homemaker / other inactive | 10,3% |

**Table 5.** Sample distribution by respondents’ education level

| Level of education | Distribution |
| --- | --- |
| No formal qualification | 1,5% |
| Primary school certificate | 1,5% |
| Lower secondary school certificate (Brevet) | 2,7% |
| Vocational certificate (CAP/BEP) | 15,7% |
| Upper secondary school diploma (Baccalaureate) | 26,5% |
| Two-year post-secondary degree (Bac +2) | 21,2% |
| Bachelor’s / Master’s level 1 (Bac +3 / Bac +4) | 15,8% |
| Master’s degree (Bac +5) | 8,7% |
| Above Master’s level / Doctorate | 6,2% |
| Don’t know / Prefer not to answer | 0,3% |

**Table 6.** Sample distribution by region

| Region | Distribution |
| --- | --- |
| Paris Region | 18,0% |
| North-West | 24,0% |
| North-East | 23,5% |
| South-West | 11,8% |
| South-East | 22,7% |

**Table 7.** Sample distribution by respondents’ income bracket

| Income bracket | Distribution |
| --- | --- |
| < €900 | 6,3% |
| €900–€1,499 | 15,8% |
| €1,500–€2,499 | 26,8% |
| €2,500–€3,499 | 24,5% |
| €3,500–€4,499 | 16,5% |
| > €4,500 | 10,0% |

***Table 8.*** *Frequency of consumption of PDO-labelled food products*

| Frequency of consumption | Distribution |
| --- | --- |
| At least once a day | 5,0% |
| 1 to 6 times per week | 30,7% |
| 1 to 3 times per month | 39,8% |
| Less than once per month | 15,2% |
| Never | 2,2% |
| I don’t know | 7,2% |
